# Intestinal Absorption Profile of Three Polygala Oligosaccharide Esters in Polygalae Radix and the Effects of Other Components in Polygalae Radix on Their Absorption

**DOI:** 10.1155/2019/1379531

**Published:** 2019-07-02

**Authors:** YinYing Ba, MengLin Wang, KunFeng Zhang, QiJun Chen, JiaJia Wang, Hang Lv, YanYan Jiang, Renbing Shi

**Affiliations:** ^1^School of Traditional Chinese Medicine, Capital Medical University, No. 10, Toutiao, You-An-Men-Wai Street, Beijing 100069, China; ^2^School of Chinese Pharmacy, Beijing University of Chinese Medicine, No. 11, Beisanhuan Dong Street, Beijing 100013, China

## Abstract

Oligosaccharide esters, which are among the main active components of Polygalae Radix (PR), demonstrate significant pharmacological activities in the human nervous system. In our previous research, some other constituents in PR were able to improve the bioavailability of oligosaccharide esters such as sibiricose A5 (SA5), sibiricose A6 (SA6), and 3,6′-disinapoyl sucrose (DISS), but the related components and their underlying mechanisms remain unknown. The present study aimed to investigate the intestinal absorptive profile of SA5, SA6, and DISS and the absorptive behavior influenced by the coadministration of polygalaxanthone III and total saponins of PR (TS) using an* in vitro* everted rat gut sac model, along with the possible mechanisms that may influence absorption. The results showed that TS could significantly enhance the absorption of SA5, SA6, and DISS monomers. Verapamil, a P-glycoprotein inhibitor, was able to elevate the absorption of SA5 and SA6, and an absorption experiment using Rho123 led us to conclude that TS influenced the absorption of SA5 and SA6 in a manner similar to that of a P-glycoprotein inhibitor. Sodium caprate, a paracellular absorption enhancer, was found to increase the absorption of SA5, SA6, and DISS. Results showed that the absorption mechanisms of SA5 and SA6 may combine active transport with paracellular passive penetration, while DISS's absorption was dominated by paracellular passive penetration. However, the relationship between polygala saponins and the absorption of SA5, SA6, and DISS by paracellular passive penetration remain to be examined. This is the direction of our future research.

## 1. Introduction

Polygalae Radix (PR, Yuanzhi), one of the commonly used traditional Chinese medicines (TCM), is derived from the roots of* Polygala tenuifolia* Willd. or* Polygala sibirica* L. It has been used for many years for the treatment of amnesia, neurasthenia, palpitation, insomnia, and depression [1]. Pharmacological research has revealed that the therapeutic effect of PR works through multiple channels and multiple pathways, such as suppressing the secretion of *β*-amyloid (*Aβ*) [2], antioxidation [3], antidepression [4–6], and neuroprotection [7, 8]. The major active constituents of PR include oligosaccharide esters, saponins, and xanthones, which have been reported to be related to the therapeutic effect of PR on diseases of the central nervous system [9–11]. Among these ingredients, the most representative ones found at the highest amounts in PR are the oligosaccharide esters. Recent studies have shown that oligosaccharide esters have significant effects on neuroprotective and antioxidant systems. They also cause improvements in the function of the central cholinergic system [12–15]. Sibiricose A5 (SA5), sibiricose A6 (SA6), and 3,6′-disinapoyl sucrose (DISS) are the main components of oligosaccharide esters [16]. These three oligosaccharide esters have been shown to have protective effects in neurotoxicity. Moreover, previous studies demonstrated that sibiricose A5 could protect PC12 cells from the damage induced by glutamic acid. DISS played an antidepressant effect through regulating the levels of brain monoamine neurotransmitters or improving the function of the hypothalamic-pituitary-adrenal (HPA) axis [17–19]. Other studies also showed that DISS is effective for depression by helping activate the phosphorylation of cAMP-response element binding protein (CREB) in the hippocampus and promoting downstream BDNF expression [20, 21]. In addition, DISS is a marker compound for RP according to the Pharmacopoeia of the People's Republic of China (2015). In our previous research, we conducted pharmacokinetic studies on SA5, SA6, and DISS following oral administration of either unpurified extract of PR (PRE) or else pure SA5, SA6, or DISS. By comparing the area under the curve (AUC) of the drug, we found that some other constituents in PRE may promote the absorption of these oligosaccharide esters and subsequently improve their bioavailability [22, 23]. However, which type of components promoted the absorption of oligosaccharide esters and the underlying mechanisms remain unknown.

**Figure 1:**
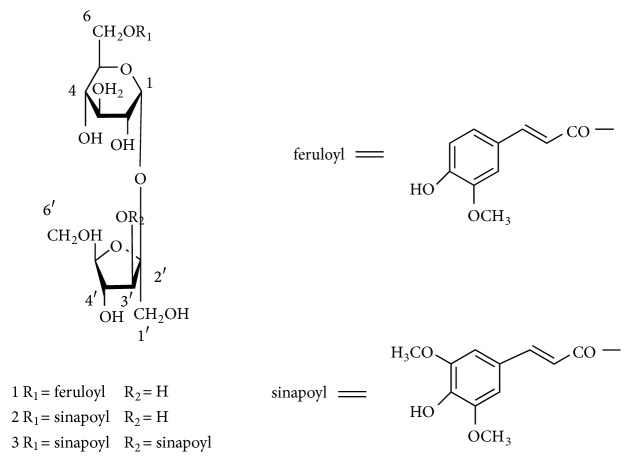
Structures of sibiricose A5, sibiricose A6, and 3,6′-disinapoyl sucrose.

The efficacy of TCM is based on the active ingredients, and the various effective components are combined in proportion to exert efficacy. With the further development of modern studies on TCM, development of modern Chinese herbal drugs by studying compatibility of various effective components in single Chinese medicines or compounds has become a key research direction. Studies on the synergy between multiple ingredients in one type of TCM could also help to reveal the mechanisms of the integral effects of any particular TCM. Therefore, in order to explore the synergistic mechanism between multiple components in RP from the point of intestinal absorption, the present research was designed to study the intestinal absorptive profile of three oligosaccharide esters (SA5, SA6, and DISS, Figure 1) in PR both in an oligosaccharide state and in a monomeric state by using a developed everted rat gut sac model* in vitro*. Then, the influence of polygalaxanthone III (PT) and total saponins (TS) on the absorptive profile of SA5, SA6, and DISS and their possible influence mechanism were investigated. Samples were collected from the sac contents and were analyzed for three oligosaccharide esters using RP-HPLC.

## 2. Materials and Methods

### 2.1. Materials

The roots of* P. tenuifolia* were purchased from Good Agricultural Practices (GAP) Base in Heyang City, Shaanxi Province. The roots were identified by Yuting Chen (Beijing University of Chinese Medicine). Sibiricose A5 and sibiricose A6 were isolated from* Polygala tenuifolia* in our laboratory [16]. Their structures were elucidated based on their spectral data (IR, MS, ^1^H-NMR, and ^13^C-NMR) and their purity was determined by HPLC (purity ≥ 96%). Polygalaxanthone III (purity ≥ 98%) was purchased from Nanchang Beta Biological Technology Co., Ltd. (Nanchang, Jiangxi Province, China). We purchased 3,6′-disinapoyl sucrose and verapamil from the National Institutes for Food and Drug Control, Beijing, China (purity ≥ 96%). Rhodamine 123 (Rho123) and sodium caprate were obtained from Sigma Chemical Co. (St. Louis, MO, USA). The culture solution was a Krebs–Ringer (K–R) culture solution, containing 3.90 g NaCl, 0.175 g KCl, 0.685 g NaHCO_3_, 0.01 g MgCl_2_, 0.16 g NaH_2_PO_4_, and 0.35 g glucose in 500 mL distilled water. MCI GEL CHP20P resin was purchased from Mitsubishi Chemical Corporation (Japan).

HPLC-grade acetonitrile and methanol were obtained from Fisher Scientific Inc. (Emerson, IA, USA). Purified water was purchased from Hangzhou Wahaha Group Co., Ltd. (Hangzhou Zhejiang Province, China). Oxygen-generating agent was purchased from Oxygen Stand Firm (Beijing, China). All of the other chemicals were of analytical grade.

### 2.2. Animals

SD rats (male, 220–260 g) were obtained from the Vital River Experimental Animal Co., Ltd. (Beijing, China). The experiments on animals were in conformity with the Guidelines of “Principles of Laboratory Animal Care” (NIH publication no. 80-23, revised 1996) and were approved by the Animal Experimentation Ethics Committee of Capital Medical University. The rats were housed in a temperature- and humidity-controlled room (25 degrees centigrade, 55% air humidity) with standard diet and water. Prior to the experiments, the rats were acclimatized for three days and had free access to water but no food for 12 h before the experiments.

### 2.3. Preparation of Drug

PR (400 g) was refluxed with water (1:10, w/v) for 2 h twice. Afterwards, removal of the solvent under reduced pressure was performed, resulting in an extract of Polygalae Radix (PRE) weighing 143.12 g. The PRE, which was analyzed by LC, contained 0.417% SA5, 0.716% SA6, 1.65% DISS, and 0.099% PT. PRE (71.56 g) was further applied to column chromatography on MCI GEL CHP20P resin eluting with H_2_O, 40% EtOH, and 80% EtOH to afford three fractions. The 80% EtOH fraction was concentrated under reduced pressure to obtain the extract of TS from Polygalae Radix (9.40 g).

### 2.4. Detection Methods

Regarding the detection of SA5, SA6, and DISS, the HPLC system consists of a Waters 2695 HPLC pump, a Waters ASI-120 automated sample injector, and a Waters 2996 Photodiode array detector (Waters, Milford, MA, USA). Samples were separated on a Waters Xbridge™ shield RP18 column (5 *μ*m, 250 mm × 4.6 mm) protected with an Xbridge™ RP18 guard column (5 *μ*m, 12.5 mm × 4.6 mm). The mobile phase consisted of CH_3_CN (A) and water containing 0.1% (v/v) phosphoric acid (B). A gradient program was used as follows: 8%–14% A (0-15 min), 14%–18% A (15–25 min), 18%–21% A (25–35 min), 21%–25% A (35–40 min), 25%–27% A (40–46 min), 27%–30% A (46–53 min), 30%–35% A (53–62 min), 35%–40% A (62–75 min), and 40%–55% A (75–85 min). The flow rate was 1.0 ml/min. The column temperature was 30°C. The detection was performed at a wavelength of 330 nm. PRE was dissolved in K-R culture solution at concentration of 2.07 mg/ml. Working standard solutions of SA5, SA6, and DISS in the concentration ranges of 1.09–130.20 *μ*g/ml, 2.25–270.00 *μ*g/ml, and 2.66–212.80*μ*g/ml, respectively, were freshly prepared in K-R culture solution. A calibration plot was obtained by plotting SA5, SA6, and DISS peak areas against concentration; a regression equation was obtained by linear regression analysis. The intraday precision was determined within one day by analyzing six replicates of samples of sac contents at concentrations of 50 *μ*g/ml (SA5), 86 *μ*g/ml (SA6), and 198 *μ*g/ml (DISS). The interday precision was determined on the third day of analysis for the samples of sac contents. The intraday and interday precision was defined as the relative standard deviation (RSD). The stability of PRE samples at room temperature for 24 h was determined six times for the sac contents. The average recoveries were determined by analyzing the PR sample solutions mixed with corresponding referenced standard six times, and we took the average expressed as a percentage value (% accuracy = [measured concentration/nominal concentration] ×100%).

For determination of Rho123, it was prepared in K–R culture solution at the concentrations of 0.001, 0.005, 0.01, 0.05, 0.1, 0.5, and 1.5 *μ*mol/L. A Varioskan Flash automatic microplate reader (Thermo, USA) was used for the detection of Rho123 analysis at an excitation wavelength of 500 nm and an emission wavelength of 525 nm. The regression equation was* Y *= 298.34*X* - 2.0279;* r* = 0.9991.

Regarding identification of chemical components in TS, the analysis was performed on an Agilent 1260 series (Agilent, USA) coupled with a Bruker FT-ICR-MS solariX MALDI/ESI 9.4T (Bruker, USA). Samples were separated on an Agilent Poroshell 120 SB-C18 column (2.7 *μ*m, 150 mm × 4.6 mm). The mobile phase consisted of CH_3_CN (A) and water (B). A gradient program was used as follows: 5%–12% A (0–5min), 12%–25% A (5–10 min), 25% A (10–18 min), 5%–30% A (18–23 min), 30%–45% A (23–33 min), and 45%–70%A (33–40 min). The flow rate was 0.4 ml/min. The column temperature was 25°C. The detection was performed at a wavelength of 330 nm. The mass spectrometer was operated in negative ion mode. The capillary voltage was 5 kV. The dryer flow rate was 6 L/min and the dryer pressure was 2.0 bar at 180°C. The balance plate effect was -500 V.

### 2.5. Drug Stability and the Studies of Sampling Time

The K–R culture solution was circulated in rat intestines for 3 h. PRE, SA5, SA6, and DISS were dissolved in the blank intestinal circulation fluid at concentrations of 12 mg/ml, 0.050 mg/ml, 0.086 mg/ml, and 0.198 mg/ml, respectively, and incubated at 37°C for 3 h. Aliquots of 1 ml samples were taken out at 0, 0.5, 1, 2, and 3 h after preparation for analysis.

### 2.6. Intestinal Absorption Experiment* In Vitro* by the Everted Rat Gut Sac Model

#### 2.6.1. Preparation of Drug Solutions

During the* in vitro* gut sac experiments, PRE (6.00 mg/ml, 12.00 mg/ml, and 18.00 mg/ml), SA5 (0.025 mg/ml, 0.050 mg/ml, and 0.075 mg/ml), SA6 (0.043 mg/ml, 0.086 mg/ml, and 0.130 mg/ml), DISS (0.099 mg/ml, 0.198 mg/ml, and 0.296 mg/ml), PT (0.012 mg/ml), and TS (1.58 mg/ml) were dissolved in the intestinal culture solution as test solutions. The medium concentration of PRE was equal to the human clinical dose. The concentrations of SA5, SA6, DISS, and PT were calculated by equal amount of PRE. The concentration of TS was calculated according to the amount of raw drugs of PRE.

#### 2.6.2. Experimental Setup [24]

The rats were anesthetized with an intraperitoneal injection of 2% phenobarbital sodium (0.4 mg/kg). After the abdominal area had been shaved, we opened the abdominal cavity with a midline incision (2–3 cm). The jejunum segment of the intestinal sac was isolated carefully and then flushed with ice-cold K-R solution immediately. We removed the underlying mesentery. The intestinal segments were cut into 10 cm lengths, everted carefully with a smooth glass rod, and tied at the outlet at one end. Then, 2 ml of drug-free K-R solution was injected into the serosa compartment and the other end was tied. The everted intestinal sac was suspended in the experimental test solution (25 ml) and maintained at 37°C. The experimental solution was continually aerated with 5% CO_2_ and 95% O_2_. Aliquots of 1 ml of serosal solution were taken out from the intestinal sac at different time intervals (0.25, 0.75, 1.25, 2, 2.5, and 3 h), and 1 ml drug-free culture solution was injected into the intestinal sac to complement the solution. After sampling, the length and width of the intestinal segments were measured. Then uptake per unit area was calculated.

#### 2.6.3. Sample Preparation

The samples were mixed with acetonitrile at a ratio of 1:0.2, vortex-mixed for 2 min, and centrifuged at 9,000 rpm for 5 min. The supernatant was obtained and filtered through a 0.45 *μ*m Millipore membrane for HPLC analysis.

#### 2.6.4. Parameter Calculation

The cumulative absorption of drugs is described by the equation* Q = 2CnVn + *∑_*i*=1_^*n*−1^*CiVi*. The sampling time was used for linear regression of the absorbed dose per unit area, and the absorption rate constant *K*_*a*_ was obtained from the slope. The apparent permeability coefficient (*P*_*app*_) is calculated as *P*_*app*_ = △*Q*/△*t*/*A*/*C*_0_, where Δ*Q* is the amount of drug permeated in Δ*t* (*μ*g), *A* is the area of the intestinal membrane, and *C*_0_ is the initial concentration. Percentage of absorption (*P*%) = drug concentration in the intestinal sac/drug concentration in the bath × 100%.

#### 2.6.5. Statistical Analyses

All of the values are expressed as the mean ±* SD* and are analyzed by one-way analysis of variance (ANOVA) and LSD test using SPSS ver. 16.0 software.* P* values lower than 0.05 were accepted as statistically significant.

## 3. Results and Discussion

### 3.1. Validation of the HPLC Methods for Detection of SA5, SA6, and DISS

**Figure 2:**
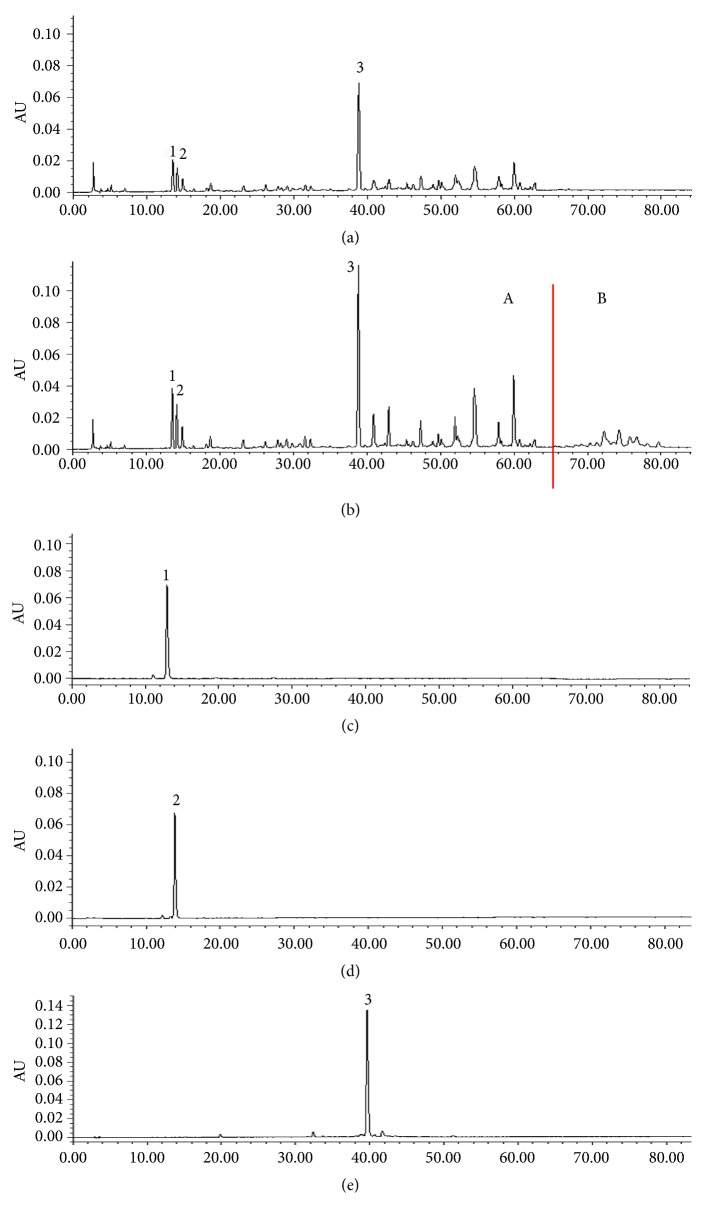
HPLC chromatograms of intestinal circulation solution of PRE (a), PRE solution (b), SA5 reference solution (c), SA6 reference solution (d), and DISS reference solution (e).

Representative chromatograms obtained from PRE solution, the sac absorption solution of PRE, and the reference solutions of SA5, SA6, and DISS are shown in Figure 2. They all exhibited a major well-resolved peak. There was good linearity of SA5, SA6, and DISS. Their typical regression equations were* Y* = 15293X – 608.48 (*r *= 0.9997, n = 6),* Y* = 7089.4*X* - 3321.1 (*r *= 0.9999, n = 6), and* Y *= 18814*X* – 37768 (*r *= 0.9999, n = 6). Both the stability and the precision of the analytical method were below 2% (n = 6). Their mean recoveries were 97.45%, 97.67%, and 97.94%. These good results showed that the method was both stable and suitable for continuous monitoring of SA5, SA6, and DISS. According to the literature [25], the fingerprint of PRE (Figure 2(b)) is roughly divided into two regions, based on the type of chemical compositions. The first region, 0 to 65 min (region A), is the mixed region of oligosaccharide esters and xanthones, and the second, after 65 min (region B), is the region of saponins. In the fingerprint of the sac absorption solution of PRE (Figure 2(a)), polygala saponins were poorly absorbed.

### 3.2. Drug Stability and Absorption Studies

The stabilities of SA5, SA6, and DISS in the blank intestinal circulation solution were RSD = 1.70%, RSD = 0.87%, and RSD = 1.96%, respectively, which showed that they were stable at 37°C for 3 h. This study was designed to ensure that the loss of the drug during the absorption experiment was due only to absorption and not other means.

### 3.3. Identification of Chemical Components in TS

**Figure 3:**
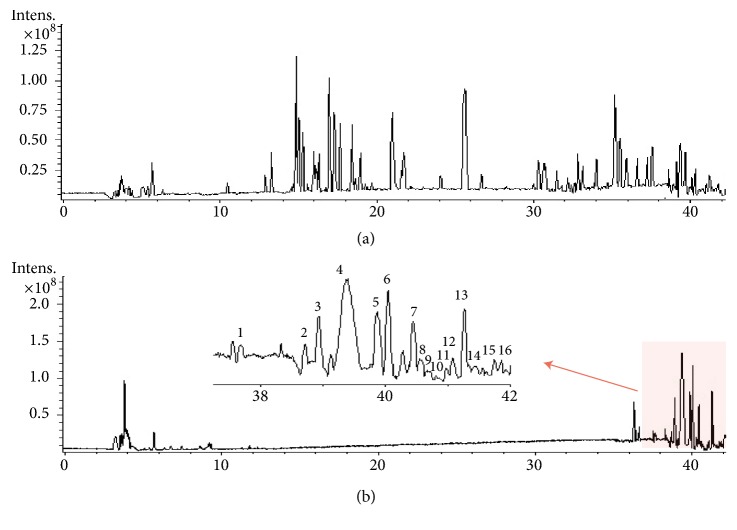
Total ion chromatograms of RP extracts (a) and TS (b) in negative ion mode.

In the present study, the total saponins from Polygalae Radix (TS), which mainly contains polygala saponins analyzed by HPLC-FT-ICR/MS, were prepared.  Figure 3 shows that TS mainly contains polygala saponins. According to the mass spectrometric data and reported literature [26, 27], 16 polygala saponins were characterized from TS (Table 1).

### 3.4. The Intestinal Absorptive Profile of SA5, SA6, and DISS Monomers

**Figure 4:**
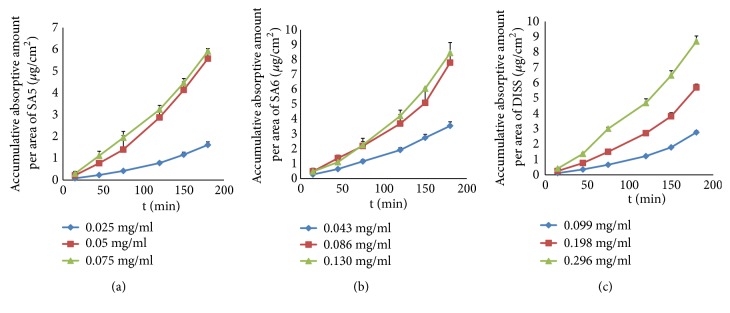
The absorption profile of SA5 (a), SA6 (b), and DISS (c) monomers. The data are presented as the mean ± SD (n = 5).

As shown in Figure 4, the accumulation of three oligosaccharide monomers increased linearly (*r *> 0.9) with time in three concentrations in the everted rat gut sac model. However, there was no statistical difference in *K*_*a*_ of SA5 and SA6 between medium and high concentrations, and the intestinal absorptive profiles of the two concentrations were similar. Moreover, *P*_*app*_ and* P%* of SA5 and SA6 in the small intestine presented a trend of increasing first and then decreasing, which is different from the characteristic of that in PRE (Table 2). These results suggested that the absorption of SA5 and SA6 would be saturated as the concentration increased, which would mean that the absorption mechanism of SA5 and SA6 monomers may involve active transport in addition to passive diffusion. Unlike SA5 and SA6, the accumulation and *K*_*a*_ of DISS increased linearly (*r *> 0.9) with concentration, and *P*_*app*_ and* P%* of DISS were unaffected by concentration. The phenomenon suggested that the absorption mechanism of DISS monomer may involve passive diffusion.

### 3.5. The Intestinal Absorptive Profile of SA5, SA6, and DISS in PRE

**Figure 5:**
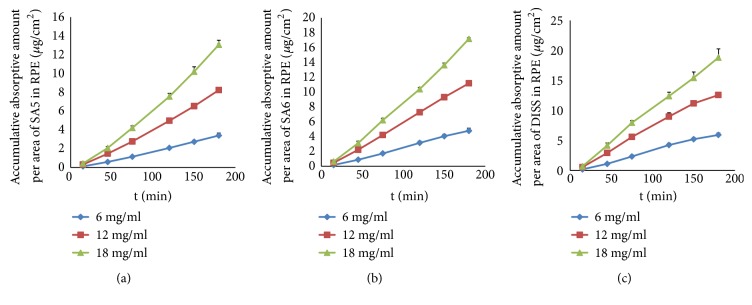
The absorptive profile of SA5 (a), SA6 (b), and DISS (c) in PRE. The data are presented as the mean ± SD (n = 5).

In this study, three different concentrations of PRE were used to investigate the intestinal absorption profile of SA5, SA6, and DISS. As shown in Figure 5, the accumulation of three oligosaccharide esters increased linearly (*r *> 0.9) with both concentration and time in low, medium, or high concentrations of PRE in the everted rat gut sac model.  Table 3 shows the absorption parameters of three oligosaccharide esters in three doses over 180 min. By comparison with the absorption parameters of SA5, SA6, and DISS in PRE, it was found that* K*_a_, *P*_*app*_, and* P%* of SA5, SA6, and DISS monomers are all significantly (*P *< 0.01) less than that of PRE-treated gut sacs in the three concentrations. These phenomena were consistent with the results of the pharmacokinetic study. Notably, and different from what we observed with SA5 and SA6 monomers,* K*_a_'s of SA5 and SA6 in PRE at high concentrations were higher (*P *< 0.01) than those at medium concentrations. For SA5 and SA6, *P*_*app*_ was significantly increased in both medium and high concentrations compared to their *P*_*app*_ in low concentrations, but there was no significant difference between *P*_*app*_ at medium and high concentrations. *P*_*app*_ of DISS in PRE remains basically unchanged with increasing drug concentration. These results suggested that the absorption enhancement of SA5, SA6, and DISS in PRE and the difference of absorptive profile between the three monomers in PRE versus these monomers alone may be related to the changes in intestinal microenvironment in rat gut induced by other chemicals in the PRE. However, the related ingredients and the underlying mechanism have so far been unclear.

### 3.6. Influence of PT or TS on the Absorptive Profile of SA5, SA6, and DISS

**Figure 6:**
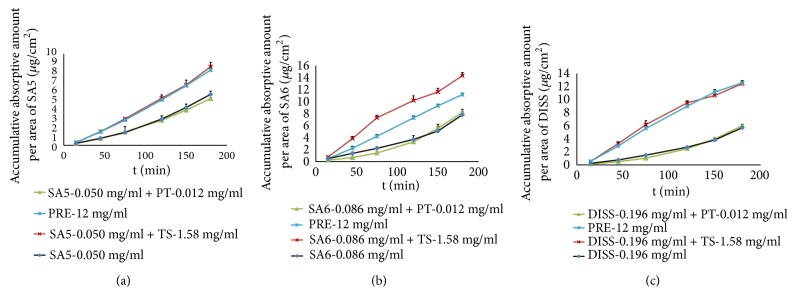
Influence of PT and TS on the absorption in the intestine of SA5 (a), SA6 (b), and DISS (c) monomers. The data are present as the mean ± SD (n = 5).

Apart from oligosaccharide esters, xanthones and triterpenoidal saponins are also main components of PR. PT, a marker compound for PR in the Pharmacopoeia of the People's Republic of China (2015), is the major compound of xanthones in PR. Polygala saponins have many pharmacological activities, including antidepression, neuroprotection, and antidementia [28–30]. All of the polygala saponins are pentacyclic triterpenoid saponins, and their basic nucleus is oleanolic acid. In the present study, SA5, SA6, and DISS at medium concentrations were coadministrated separately with PT (0.012 mg/ml) or TS (1.58 mg/ml) for 180 min to examine the effects of PT or TS on the absorptive profile of the three oligosaccharide esters. When SA5 (0.050 mg/ml), SA6 (0.086 mg/ml), and DISS (0.198 mg/ml) were coincubated with PT, the results (shown in Figure 6) suggested that there was no significant increase or decrease in the three oligosaccharide esters' absorption in gut sacs after treatment with PT. This suggested that these three oligosaccharide esters' absorption was not influenced by PT in rat intestine. However, as Figure 6 shows, when TS (1.58 mg/ml) was added to the K–R culture solution containing SA5, SA6, and DISS, respectively, the absorption of SA5 (0.050 mg/ml), SA6 (0.086 mg/ml), and DISS (0.198 mg/ml) significantly increased (*P *< 0.01). The absorptive profiles of SA5 and DISS were close to that of the PRE incubated gut sacs over 180 min, while the absorption kinetic parameters such as* K*_a_ and *P*_*app*_ of SA6 cotreated with TS were higher (*P *< 0.01) than those of the PRE's. That is, when these three oligosaccharide esters were coincubated with TS, the cumulative amount per intestinal area of them was 1.59, 1.84, and 2.18 times more than that treated with monomers alone at 180 min. There were statistically significant differences in these measurements (*P *< 0.01). This showed that although the absorption of polygala saponins was poor (Figures 2(a) and 2(b)), they may enhance the absorption of these oligosaccharide esters. To our knowledge, this is the first report of these phenomena.

### 3.7. Influences of Verapamil on the Absorptive Profile of SA5, SA6, and DISS

**Figure 7:**
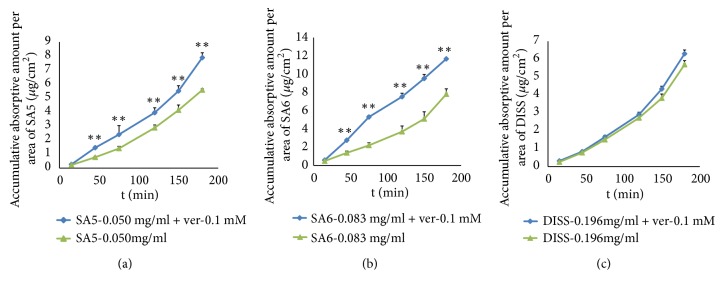
Influence of verapamil (ver) on the absorption in the intestine of SA5 (a), SA6 (b), and DISS (c) monomers. The data are presented as the mean ± SD (n = 5); ^**∗**^*P* < 0.05 and ^**∗∗**^*P* < 0.01, compared with non-verapamil-treated gut sacs.

In addition to providing information on absorption mechanisms of drugs, the everted gut sac model was also useful to explore drug interactions with the ATP binding cassette transporter proteins including P-glycoprotein (P-gp), which is enriched in the small intestine. P-gp may reduce the absorption of P-gp substrate drugs. Inhibition of P-gp's function or decreasing its expression by use of a P-gp inhibitor like verapamil could improve the bioavailability of P-gp substrate drugs [31]. In order to determine the possible effect of intestinal P-gp on the intestinal absorption of the three oligosaccharide esters, SA5 (0.050 mg/ml), SA6 (0.086 mg/ml), and DISS (0.198 mg/ml) were coincubated with the P-gp inhibitor verapamil (0.1 mmol/L). The results showed the cumulative absorbed amount of SA5 and SA6 per intestinal area increased up to about 1.41–1.50-fold (P < 0.01) at 180 min by combination with verapamil (Figure 7). In addition, the absorption rates (*K*_a_) of SA5 and SA6 were 0.044 ± 0.0077 *μ*g·cm^2^·min (n = 5) and 0.066 ± 0.010 *μ*g·cm^2^·min (n = 5) faster (P < 0.01) than that of non-verapamil-treated gut sacs. In 180 min, verapamil increased *P*_*app*_ from (1.34 ± 0.092) × 10^−5^ to (1.45 ± 0.093) × 10^−5^cm/s for SA5 and from (0.80 ± 0.079) × 10^−5^ to (1.27 ± 0.034) × 10^−5^cm/s for SA6 with a significant difference (p < 0.01). This indicated that the intestinal transportation and absorption of SA5 and SA6 could be markedly enhanced along with the inhibition of P-gp induced by verapamil and the bioavailability of SA5 and SA6 could subsequently be increased. However, there was no significant influence on 3,6′-disinapoylsucrose transport by verapamil, which suggested that DISS was not a substrate for P-gp. This is consistent with the results of Chen et al. [32].

### 3.8. Influences of Sodium Caprate on the Absorption Profile of SA5, SA6, and DISS

**Figure 8:**
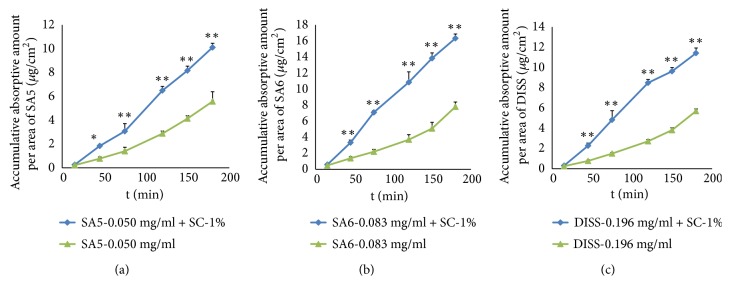
Influence of sodium citrate (SC) on the absorption in the intestine of SA5 (a), SA6 (b), and DISS (c) monomers. The data are presented as the mean ± SD (n = 5); ^**∗**^*P* < 0.05 and ^**∗∗**^*P* < 0.01, compared with non-verapamil-treated gut sacs.

The literature [32] states that there was no significant effect on DISS absorption with treatment with the P-gp inhibitor cyclosporine A, and the ATP energy inhibitor sodium azide was observed to have no effect either. However, coadministration with paracellular enhancers such as EDTA and sodium caprate (SC) could increase *P*_*app*_ of DISS, which means that DISS might be transported across the intestinal mucosa by paracellular passive penetration. In the present study, sodium caprate (1%) was coadministered with SA5 (0.050 mg/ml), SA6 (0.086 mg/ml), and DISS (0.198 mg/ml) to observe the possible influences on the intestinal absorption of the three oligosaccharide esters. The cumulative absorption amount of these esters per area increased up to about 1.87-, 2.08-, and 2.00-fold, respectively (P < 0.01), at 180 min by combination with SC (Figure 8). In addition, the absorption rates (*Ka*) of the three oligosaccharide esters were 0.061 ± 0.0077 *μ*g/cm^2^/min (n = 5), 0.0963 ± 0.010 *μ*g·cm^2^·min (n = 5), and 0.069 ± 0.0031 *μ*g·cm^2^·min (n = 5) faster, respectively (P < 0.01), than that of monomers alone. These results suggested that SC could significantly enhance and increase the absorption of SA5, SA6, and DISS in the small intestine. The increased absorption might indicate that the absorption mechanism of SA5 and SA6 combined active transport with paracellular passive penetration, while the absorption mechanism of DISS was dominated by paracellular passive penetration.

### 3.9. Influence of TS on the Absorption Profile of Rho123

**Figure 9:**
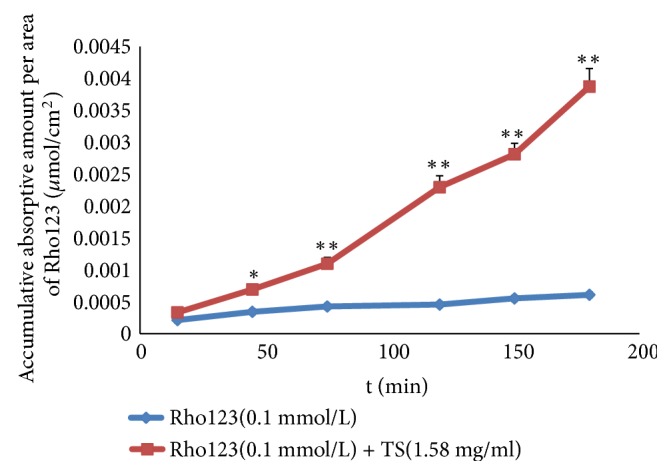
Influence of TS on the absorption of P-gp substrate Rho123 in the everted rat gut sac system. The data represent the means ± SD (n = 5); ^**∗**^*P* < 0.05 and ^**∗∗**^*P* < 0.01, compared with Rho123 alone.

Rho123 is a well-known substrate for P-gp, whose absorption can be significantly enhanced by P-gp inhibitors, such as verapamil. [33]. Therefore, Rho123 was coadministred with TS (1.58 mg/ml) in the everted rat gut sac system to investigate whether TS could increase the intestinal absorption of P-gp substrate-like drugs. The results demonstrated that the absorption of Rho123 by combination with TS was significantly enhanced (P < 0.01). The Rho123 absorption was increased about 6.35-fold at 180 min in comparison with that in the medium containing Rho123 alone (Figure 9). This indicated that TS can influence Rho123 absorption as verapamil does, and TS can enhance the intestinal absorption of SA5 and SA6 via inhibition of P-gp activity. To our knowledge, this is the first report of the P-gp substrate-like property of SA5 and SA6 and the P-gp inhibitor-like attribute of TS. However, the growth mechanisms of absorption of these oligosaccharide esters cannot be fully explained. Paracellular passive penetration, one of the absorption mechanisms of SA5 and SA6, was the dominant absorption mechanism of DISS. It is unclear how the polygala saponins affect the absorption of the three oligosaccharide esters in paracellular passive penetration. As saponin components, polygala saponins have a surfactant-like action. It has been reported that some surfactants or saponins like saikosaponin could open up intercellular tight junctions of intestinal epithelial cells, thereby promoting drug absorption [34]. Further research should be carried out to illustrate the ability of polygala saponins to open up intercellular tight junctions among cells by determination of the electrical resistance of Caco-2 cells before and after combination of the three oligosaccharide esters with polygala saponins.

Oral administration is the most common route for TCM. It is important to investigate the intestinal absorption process of the main ingredients in TCM. Up to the present, several intestinal absorption methods or models include* in vivo*,* in vitro,* and* in situ* routes. The* in vitro* everted rat gut sac model is a simple and efficient method to study the permeability and absorption kinetics of oral drugs to provide information on absorption mechanisms of drugs. Compared with other* in vitro* methods, it has an advantage for analytical experiments because the volume in the intestinal sac is small, and the concentration of a drug can be relatively high [35]. In the present study, we also found that the everted rat gut sac model was a useful and quick method for research on the interactions of various compounds.

## 4. Conclusion

In conclusion, by using the everted gut sac model, the overall absorption profile of SA5, SA6, and DISS can be enhanced by combination with TS, which are other effective components in PR. We showed that both the P-gp inhibitor verapamil and paracellular enhancers, such as sodium caprate, could significantly enhance the absorption of SA5 and SA6. This suggests that the absorption mechanism of SA5 and SA6 combines active transport with paracellular passive penetration. Therefore, the data from the Rho123 absorption experiment led us to conclude that TS can enhance the intestinal absorption of SA5 and SA6 like a P-gp inhibitor would. But this is not the whole reason. Indeed, the absorption of DISS was affected only by sodium caprate, and its absorption was dominated by paracellular passive penetration. Further research should be carried out to understand the relationship between polygala saponins and the absorption of SA5, SA6, and DISS by paracellular passive penetration.

## Figures and Tables

**Table 1:** Identification of chemical constituents in TS by HPLC/FT-ICR-MS.

peak	t_R_	[M−H]^−^ (m/z)		Formula	Identification
	(min)	Calculated	Measured		
1	37.70	679.369 4	679. 366 5	C_36_H_56_O_12_	Tenuifolin
2	38.65	1 733.743 4	1 733.744 0	C_81_H_122_O_40_	Onjisaponin S
3	39.02	1 877.785 7	1 877.788 5	C_87_H_130_O_44_	Onjisaponin Sg
4	39.23	1 791.748 9	1 791.751 7	C_83_H_124_O_42_	Onjisaponin T
5	39.88	1 847.775 1	1 847.775 7	C_86_H_128_O_43_	Onjisaponin L
6	40.12	1 631.711 7	1 631.712 3	C_77_H_116_O_37_	Onjisaponin O
7	40.51	1 571.690 6	1 571.691 1	C_75_H_112_O_35_	Onjisaponin B
8	40.69	1 617.696 1	1 617.696 6	C_76_H_114_O_37_	Onjisaponin R
9	40.70	1 685.722 3	1 685.722 8	C_80_H_118_O_38_	Onjisaponin Ng
10	40.86	1 673.722 3	1 673.722 8	C_79_H_118_O_38_	Polygalasaponin XXXII
11	40.93	1 455.643 3	1 455.643 8	C_70_H_104_O_32_	Onjisaponin G
12	40.99	1 761.738 3	1 761.738 9	C_82_H_122_O_41_	Onjisaponin Vg
13	41.43	1 485.653 8	1 485.654 4	C_71_H_106_O_33_	Onjisaponin E
14	41.63	1 587.685 5	1 587.686 1	C_75_H_112_O_36_	Onjisaponin F/ Polygalasaponin XXXI
15	41.94	1 731.727 8	1 731.728 3	C_81_H_120_O_40_	Onjisaponin W
16	41.99	1 599.685 5	1 599.686 1	C_76_H_112_O_36_	Onjisaponin Gg

**Table 2:** Absorption parameters of SA5, SA6, and DISS in the intestines of rats ($\bar{x}\pm s$; n = 5).

Compound	Concentration (mg/ml)	*K*_*a*_ (*μ*g·cm^−2^·min^−1^)	*P*_*app*_ (×10^−5^cm/min)	*P* (%)
SA5	0.025	0.0091 ± 0.0011	0.61 ± 0.070	2.59 ± 0.23
	0.050	0.031 ± 0.0012*∗∗*	1.05 ± 0.039 ^*∗∗*^	4.33 ± 0.13 ^*∗∗*^
	0.075	0.033 ± 0.0014*∗∗*	0.73 ± 0.032 ^*∗*,##^	3.16 ± 0.07 ^*∗*,##^
SA6	0.043	0.020 ± 0.0017	0.77 ± 0.065	3.29 ± 0.25
	0.086	0.041 ± 0.0041*∗∗*	0.80 ± 0.079	3.64 ± 0.27
	0.130	0.048 ± 0.0040*∗∗*^,^	0.62 ± 0.52 ^*∗*,#^	2.79 ± 0.11 ^*∗*,##^
DISS	0.099	0.015 ± 0.0005	0.26 ± 0.068	1.13 ± 0.05
	0.198	0.032 ± 0.0015*∗∗*	0.27 ± 0.013	1.15 ± 0.04
	0.296	0.049 ± 0.022 ^*∗∗*##,^	0.28 ± 0.079	1.18 ± 0.04

^**∗∗**^ *P* < 0.01 and ^**∗**^*P* < 0.05, compared with low dose; ^##^*P* < 0.01 and ^#^*P* < 0.05, compared with medium dose.

**Table 3:** Absorption parameters of SA5, SA6, and DISS in PRE in the intestines of rats ($\bar{x}\pm s$; n = 5).

Compound	Concentration (mg/ml)	*K*_*a*_ (*μ*g·cm^−2^·min^−1^)	*P*_*app*_ (×10^−5^cm/s)	*P* (%)
SA5	0.025	0.020 ± 0.0013	1.34 ± 0.092	5.44 ± 0.42
	0.050	0.048 ± 0.0011 ^*∗∗*^	1.61 ± 0.035 ^*∗∗*^	6.60 ± 0.17 ^*∗∗*^
	0.075	0.077 ± 0.0030 ^*∗∗*,##^	1.71 ± 0.067 ^*∗∗*,^	6.98 ± 0.25 ^*∗∗*^
SA6	0.043	0.029 ± 0.0020	1.12 ± 0.077	4.46 ± 0.33
	0.086	0.066 ± 0.0080 ^*∗∗*^	1.27 ± 0.093 ^*∗*^	5.21 ± 0.12 ^*∗*^
	0.130	0.100 ± 0.0067 ^*∗∗*,##^	1.29 ± 0.086 ^*∗∗*^	5.34 ± 0.04 ^*∗∗*^
DISS	0.099	0.036 ± 0.0019	0.61 ± 0.0033	2.55 ± 0.03
	0.198	0.075 ± 0.0013 ^*∗∗*^	0.63 ± 0.011	2.71 ± 0.12
	0.296	0.109 ± 0.010 ^*∗∗*,##^	0.61 ± 0.057	2.54 ± 0.19

^**∗∗**^ *P* < 0.01 and ^**∗**^*P* < 0.05, compared with low dose; ^##^*P* < 0.01 and ^#^*P* < 0.05, compared with medium dose.

## Data Availability

The data used to support the findings of this study are available from the corresponding author upon request.
